# Rates of, and factors associated with, common mental disorders in homeworking UK Government response employees’ during COVID-19: a cross-sectional survey and secondary data analysis

**DOI:** 10.1186/s40359-024-01921-4

**Published:** 2024-08-07

**Authors:** Charlotte E. Hall, Samantha K. Brooks, Henry W.W Potts, Neil Greenberg, Dale Weston

**Affiliations:** 1 Department of Psychological Medicine, King’s College London, Weston Education Centre, London, SE5 9RJ UK; 2 Behavioural Science and Insights Unit, Evaluation & Translation Directorate, Science Group, UKHSA, Porton Down, Salisbury, SP4 0JG UK; 3 Institute of Health Informatics, University College London, London, UK

## Abstract

**Introduction:**

Working on the frontline during the COVID-19 pandemic has been associated with increased risk to mental health and wellbeing in multiple occupations and contexts. The current study aimed to provide an insight into the rate of probable mental health problems amongst United Kingdom (UK) Government employees who contributed to the COVID-19 response whilst working from home, and to ascertain what factors and constructs, if any, influence mental health and wellbeing in the sample population.

**Method:**

This paper reports on the findings from two studies completed by UK Government employees. Study 1: A cross-sectional online survey, containing standardised and validated measures of common mental health disorders of staff who actively contributed to the COVID-19 response from their own homes. Binary logistic regression was used to assess factors associated with mental health outcomes. Study 2: A secondary data analysis of cross-sectional survey data collected across three timepoints (May, June, and August) in 2020 focusing on the wellbeing of employees who worked from home during the COVID-19 pandemic.

**Results:**

Study 1: 17.9% of participants met the threshold criteria for a probable moderate anxiety disorder, moderate depression, or post-traumatic stress disorder. Younger, less resilient, less productive individuals, with lower personal wellbeing and less enjoyment of working from home, were more likely to present with poorer mental health. Study 2: Found lower wellbeing was consistently associated with having less opportunities to look after one’s physical and mental health, and having unsupportive line managers and colleagues.

**Conclusion:**

It is important to ensure UK Government employees’ psychological needs are met whilst working from home and responding to enhanced incidents. It is recommended that workplaces should be seeking to continually build and improve employee resilience (e.g., through opportunities to increase social ties and support networks), essentially ensuring employees have necessary resources and skills to support themselves and others.

**Supplementary Information:**

The online version contains supplementary material available at 10.1186/s40359-024-01921-4.

## Introduction

On the 11th of March 2020, the World Health Organisation declared a global pandemic due to a novel coronavirus, COVID-19 [1]. Within the UK, the Government put in place many behavioural interventions with the aim of reducing transmission of the virus, which highly impacted usual day to day life for the public. For example, restricting how many times, and under what conditions, an individual could leave their home, as well as highly restricting social opportunities. As of the 16th of March 2020, the public were instructed to “start working from home where possible” [2].

Nearly half of those in employment were reported to work from home the following month (April 2020; [3]), a vast increase in comparison to pre-pandemic estimates of around 5% [4]. Prior to the COVID-19 pandemic, research surrounding working from home was mixed in impact. For some, it was often seen as advantageous (e.g., in terms of decreasing or eliminating commuting time; [5]) and for others more challenging (e.g., in relation to blurred boundaries between work and home life; [6]).

### Working from home during and post COVID-19

More recent systematic reviews of literature (i.e., post 2020) have established that working from home can have a mixed impact on mental health, wellbeing, productivity [7] and employee performance [8]. For example, a recent systematic review examining 27 papers (including both peer reviewed and grey literature) sought to establish whether there is an association between working from home and both mental health and productivity; specifically, for those who experienced working from home during the COVID-19 pandemic. In terms of mental health, many outcomes were examined by the included papers (e.g., but not limited to, depression, stress, psychological distress, mental wellbeing). Many of the included papers (*n* = 15) reported a negative relationship between homeworking, mental health, and wellbeing, with some concluding a mixed effect (*n* = 3) and others no effect (*n* = 2). Similar findings were also reported for productivity outcomes. The review essentially showcases that working from home can benefit some, and disadvantage others. As a result of varied findings, examination of personal and practical factors that may impact the relationship between working from home and mental health were also carried out in the review. In summary, being female, older in age, living and working in a crowded or confined home, or having young children at home were consistently associated with worsened mental health. Establishing that demographic factors and contextual factors (e.g., people in the household when working from home) may influence mental health and wellbeing outcomes.

These findings align with other research in the field that also states that variation in experiences of working from home is often reported due to a plethora of contextual and situational factors [8, 9]. To demonstrate factors associated with working from home, a recent umbrella review (i.e., review of reviews) was conducted by the current research team. The review identified a large number of apparent factors (19 in total) related to employee experience. These factors related to working environment (e.g., workplace design, space conditions), personal impact (e.g., satisfaction, career impact), and health (e.g., physical health, well-being) ([9], p.1). The review reports the majority of all derived factors to be mixed in outcome (e.g., some employees have access to appropiate space and equipment whereas others do not; some employees find working from home to positively impact their wellbeing where others do not), again confirming the variety of experiences when working from home – as home environments and employee’s personal preferences differ. Therefore, it is important to gain clarity on which factors most impact wellbeing outcomes, in order to be able to mitigate and offer support to those most as risk of poorer wellbeing when working from home.

### Challenges with working from home

The concept of working from home also raises new challenges. For example, two recent literature reviews found that isolation and lack of social connection having a negative impact on mental health and wellbeing was consistently noted across the literature [7, 9]. When working disparately, and communicating purely online, homeworking employees may lose the ability to create a shared sense of social identity with colleagues [10], the extent to which group members perceive themselves as part of a collective ‘us’ or ‘we’ (rather than ‘I’ and ‘me’). Social group membership has the capacity to serve as a ‘social cure’, often considered an independent protective factor against ill health, particularly when there is strong identification among group members [11]. However, group identification can also be considered a ‘social curse’, and hinder positive outcomes, particularly if group members do not provide levels of social support expected [12]. More generally, the importance of social support has been showcased in recent reviews [13], and has also been found to be protective of workplace stress [14] and burnout [15]. Therefore, establishing the impact of social support and identify on mental health outcomes of those working from home may aid in tailoring interventions for improving experience.

### Current research focus

In summary, the impact of working from home has been mapped in terms of mental health, wellbeing, and productivity. Consistently, mixed findings are apparent, with many reports establishing an equivocal or negative impact at best. In the context of continued mixed findings, it may be beneficial to take a job-specific approach, to potentially minimise additional extraneous factors [9]. The mental health and wellbeing impact of the COVID-19 pandemic for various occupations working on the frontline are well documented and consistently noted as negative. For example, in relation to hospital workers [7, 16] teachers [17] and social workers [18]. One group who have received less attention are civil servants. UK civil servants who were contributing to, and providing, effective delivery of the coronavirus response are considered frontline employees [19] and were thus at high risk of the combined demands of working from home and frontline demands.

More general research established that 98% of UK civil servants were working from home in July of 2020 [20]. Recent work suggests that civil servants were likely to face a series of unmet needs in relation to their remote workplace and resources due to the sudden change to the way of working and, as a result, many of the preparatory steps recommended for effective remote working (e.g., ensuring safe, comfortable and appropiate remote workplaces and technical equipment [9, 21]) could not be carried out in time [22]. Civil servants during the COVID-19 pandemic were reported to face very high job demands [23]. Additionally, a decrease in personal wellbeing (i.e., in terms of life satisfaction, happiness, anxiousness, and belief that things in life are worthwhile was also apparent from a series of Civil Service data [24].

The current research used a two-study approach to explore the wellbeing of response-focused UK Civil Servants who worked from home during the COVID-19 pandemic and were from one select government organisation. Study 1 used a cross-sectional survey to establish the rate of probable mental health problems using standardised and validated measures, and to ascertain what factors and constructs, if any, influenced mental health in the sample population. Study 2 involved secondary data analysis of three cross-sectional surveys with UK civil servants who were working from home over the COVID-19 pandemic, which sought to compliment Study 1 by providing further clarity on potential risk and resilience factors for wellbeing.

## Study 1

### Method

#### Survey design

An online cross-sectional survey was used to understand participants’ experiences and perceptions of working from home. The survey consisted of three main parts: (1) demographic and professional questions; (2) experiences and perceptions of working from home; (3) various measures relating to mental health and wellbeing, resilience, and productivity. The findings from this survey have been split into two papers (please see: [25]), the current focuses on parts one and three. Data collection occurred between 1 May and 1 August 2022. Qualtrics was used to build and host the survey, it was estimated that the questionnaire took between five to ten minutes to complete.

### Survey distribution

All participants were recruited from one select UK Government response-focused organisation. Participants were firstly recruited via UK Government team and department leads (or equivalent) acting as gatekeepers, who cascaded information about the study via an email containing a study summary, participant information sheet and the survey link. Initial plans were to collect data within one month (1st of May to 1st of June 2022), but responses were initially slow (only around 20 responses in the first month); potentially due to small or overlapping gatekeeper distribution, as well as trying to recruit a busy workforce. The survey response period was then lengthened (May 1st – August 1st), and distributed twice using an organisational weekly newsletter, which generated more responses. After conversing with the email secretary who distributes the newsletters, it is estimated that the newsletter was delivered to over ten thousand employees, with around 20% opening the email.

### Selection criteria

To take part in this research participants needed to be over the age of 18 and have experience of working from home on the UK Government on the COVID-19 response. Participants were required to have reached the end of the survey in order to be included in data analysis.

### Ethics

The current study was carried out in accordance with the British Psychological Society Code of Ethics and Conduct [26], and was approved by the King’s College London Ethics Committee (reference number: HR/DP-21/22-26693). Informed consent to participate was obtained from all participants in the study using the first page of the survey. To mitigate any pressure that may be felt by potential participants as a result of using gatekeepers, participants were assured that the gatekeeper would not know who took part in the survey. Additionally, the participant information sheet and survey both state that nobody within the organisation would know if they participated in the study or not. Participants were made aware that their participation was voluntary, and their data would be anonymised. Participants were also able to stop at any point during the survey.

### Study materials

The survey included a range of demographic and professional information, homeworking preference, mental health, wellbeing, resilience, productivity, and items related to social support. A copy of the survey is presented in Appendix 1.

### Demographic and professional information

Participants were firstly asked a filter question: ‘Have you worked on the COVID-19 response?’ and if participants answered they had not worked on the COVID-19 response, they were filtered out from the survey. For demographic factors, participants were asked for their age, ethnicity, and sex. They were also asked for the number of people living in their household and whether there were any children living in the household. For professional information, participants were asked whether they currently (at the time of completing the survey) worked on the COVID-19 response, their length of time with the organisation and pay grade.

### Homeworking preferences

To assess perception of homeworking participants were asked to answer, using a 10-point Likert scale (1 = not at all, 10 = completely), the following statement: “*I enjoy working from home*”. This measure was created by the research team. Participants were then able to indicate their future way of working preference by answering the question “*In the future*,* I would like to…*” and selecting one of the following answers: ‘*full time homework’*; ‘*full time office work’*; ‘*work from both home and the office (hybrid arrangement)*’.

### Anxiety

The 7-item Generalized Anxiety Disorder (GAD; [27]) scale was used to measure probable anxiety disorder. This scale was chosen due to the standardised and validated nature [28] of the survey. Additionally, as this scale is widely used (e.g., [16, 29, 30]) it provides the opportunity to compare across other populations and samples. The GAD uses a 4-point Likert-scale ranging from ‘Not at all’ (equalling a score of 0) to ‘Nearly every day’ (equalling a score of 3) to assess how often an individual has been bothered by various anxiety symptoms over the past two weeks, with a higher score indicating higher levels of anxiety. Assessed symptoms include: “*Not being able to stop or control worrying?*” and “*Being so restless that it is hard to sit still?*”. In the current study, a score of > 9 was coded to indicate probable moderate anxiety disorder, and a score of > 15 coded to indicate severe anxiety disorder (in line with [27]). The Cronbach’s alpha coefficient for the current study was 0.90.

### Depression

The 9-item Patient Health Questionnaire (PHQ; [31]) was used to measure probable depression. This scale was chosen due to the standardised and validated nature [31] of the survey. Additionally as this scale is widely used (e.g., [16, 29, 30, 32]) it provides the opportunity to compare across other populations and samples. The PHQ uses a 4-point Likert-scale ranging from ‘Not at all’ (equalling a score of 0) to ‘Nearly every day’ (equalling a score of 3) to assess how often an individual had been bothered by various depressive symptoms in the previous two weeks, with higher scores indicating higher levels of depression. Assessed symptoms include: “*Feeling down*,* depressed*,* or hopeless?*” and “*Trouble concentrating on things*,* such as reading the newspaper or watching television?*”. In the current study, a score of > 9 was coded to indicate probable moderate depression, and a score of > 19 coded to indicate severe depression (in line with [31]). The Cronbach’s alpha coefficient for the current study was 0.88.

### PTSD

The 6-item Post-Traumatic Checklist – Civilian Version (PCL-C; [33]) was used to measure probable PTSD. This scale was chosen due to the standardised and validated nature in nonclinical samples [34]. Additionally, as this scale is commonly used (e.g., [16, 30, 35]) it provides the opportunity to compare across other populations and samples. The PCL-C uses a 5-point Likert-scale ranging from ‘Not at all’ (equalling a score of 1) to ‘Extremely’ (equalling a score of 5) to assess how often an individual has been bothered by various problems/complaints indicative of post-traumatic stress over the past month, with higher scores indicating higher levels of post-traumatic stress. Assessed problems/complaints include: “*Feeling very upset when something reminded you of a stressful experience from the past?*” and “*Feeling irritable or having angry outbursts?*”. A score of > 17 was coded to indicate probable PTSD (in line with [36]). The Cronbach’s alpha coefficient for the current study was 0.89.

### Personal wellbeing

Participants were asked to answer the following single questions using an 11-point Likert scale ranging from 0 (not at all) to 10 (completely): (1) “*Overall*,* how satisfied are you with your life nowadays?”*, (2) “*Overall*,* to what extent do you feel that the things that you do in life are worthwhile?”*, (3) “*Overall*,* how happy did you feel yesterday?”*, (4) “*Overall*,* how satisfied are you with your job nowadays?”*. The first three listed questions are in their original form and are regularly used by the Office for National Statistics [37], and the fourth was adapted by the researcher team to assess job satisfaction. These measures are widely used to measure personal wellbeing [38] and are also concurrent with Study 2. In the current study, a score of > 6 was coded to indicate high satisfaction, happiness, or belief of a worthwhile life, in line with [37].

### Resilience

The 6-item Brief Resilience Scale (BRS; [39]) was used to measure resilience. The questionnaire was used in its original form and chosen for the ability to measure personal resilience [39] whilst also minimising participant burden. The BRS uses a 5-point Likert scale ranging from ‘Strongly disagree’ (equalling a score of 1) to ‘Strongly agree’ (equalling a score of 5) to answer a series of statements related to resilience, for example “*I have a hard time making it through stressful events*” or “*It does not take me long to recover from a stressful event*”. Three out of six statements are reverse coded. Scores were summed and an average calculated, with a higher score indicating higher levels of resilience. A score of 1.00-2.99 was categorised as low resilience, 3.00-4.30 as normal resilience, and 4.31-5.00 as high resilience, in line with [39]. The Cronbach’s alpha coefficient for the current study was 0.88.

### Job performance

The 18-item Individual Work Performance Questionnaire (IWPQ; [40]) was used to measure job performance. The questionnaire was used in its original form and was chosen due to the ability to measure individual work performance, which is particular important when employees are working from their own homes. Additionally, the questionnaire is deemed to be reliable and valid [40]. The questionnaire measures three dimensions of job performance: Task performance (e.g., “*I managed to plan my work so that it was done on time*”; TP), Contextual performance (e.g., “*I started new tasks myself*,* when my old ones were finished*”), and Counterproductive work behaviour (e.g., “*I complained about unimportant matters at work*”). The IWPQ uses a 5-point Likert-scale ranging from ‘Seldom’ (equalling a score of 0) to ‘Always’ (equalling a score of 4) for task and contextual performance, and ‘Never’ (equalling a score of 0) to ‘Often’ (equalling a score of 4) for counterproductive work behaviour. Scores are summed for each scale and an average calculated, with a higher score indicating higher levels of performance for TP and CP, and a lower score indicating less CWB. For TP, a score of up to 2.16 was categorised as low performance, 2.17–2.99 as average, and more than 3.00 as high (in line with [41]). For CP, a score of up to 1.87 was categorised as low performance, 1.88–2.87 as average, and more than 2.88 as high (in line with [41]). For CWB, a score of up to 0.79 was categorised as low levels of behaviour, 0.80–1.59 as average, and more than 1.60 as high [41]. The Cronbach’s alpha coefficient values for the current study were 0.83 (TP); 0.87 (CP); and 0.80 (CWB).

### Social support and identities

Identification with others was measured using adapted versions of two identification questions [42] using a 7-point Likert scale ranging from ‘Not at all’ (equalling a score of 1) to ‘Definitely’ (equalling a score of 7). The items were: “*I identify with others in my workplace*” and “*I feel strong ties with others in my workplace*”. Scores were summed and an average calculated, with a higher score indicating higher levels of identification with the workplace. The Cronbach’s alpha coefficient for the current study was 0.83.

Social support was measured using adapted versions of four identification questions [43] using a 7-point Likert scale ranging from ‘Not at all’ (equalling a score of 1) to ‘Definitely’ (equalling a score of 7). The items were: “*Do you get the emotional support you need from other people?”*, “*Do you get the help you need from other people?”*, “*Do you get the resources you need from other people?”* and, “*Do you get the advice you need from other people?”.* Scores were summed and an average calculated, with a higher score indicating higher levels of social support. The Cronbach’s alpha coefficient for the current study was 0.89.

Having multiple identities (i.e., sense of belonging to groups, usually associated with better adjustment and greater well-being [44]) was measured using adapted versions of four identification questions [43] using a 7-point Likert scale ranging from ‘Not at all’ (equalling a score of 1) to ‘Definitely’ (equalling a score of 7). The items were: “*Before the COVID-19 pandemic I belonged to lots of different groups*”, “*Before the COVID-19 pandemic I joined in the activities of lots of different groups*”, “*Before the COVID-19 pandemic I had friends who were members of lots of different groups*” and, “*Before the COVID-19 pandemic I had strong ties with lots of different groups*”. Scores were summed and an average calculated, with a higher score indicating higher levels of multiple identities. The Cronbach’s alpha coefficient for the current study was 0.96.

Identity continuity (i.e., sense of remaining a member of groups over time or throughout event, associated with good wellbeing in the workplace [45]) was measured using adapted versions [43] of four identification questions using a 7-point Likert scale ranging from ‘Not at all’ (equalling a score of 1) to ‘Definitely’ (equalling a score of 7). The items were: “*I still belong to the same groups I was a member of before the start of the COVID-19 pandemic*”, “*I still join in the same group activities as I did before the start of the COVID-19 pandemic*”, “*I am friends with people in the same groups as I was before the start of the COVID-19 pandemic*” and, “*I continue to have strong ties with the same groups as I did before the start of the COVID-19 pandemic*”. Scores were summed and an average calculated, with a higher score indicating higher levels of multiple identities. The Cronbach’s alpha coefficient for the current study was 0.94.

### Analysis

Descriptive statistics to describe the sample population were firstly calculated using counts and percentages. These were also used to establish the rate of probable depression, anxiety, and PTSD in the sample. Due to high rates of correlation between each of the measures of mental health (*r* ≥ .80), a binary variable coined ‘any mental disorder’ (AMD) was created to indicate presence of probable moderate anxiety disorder (as measured by the GAD-7), probable moderate depression (as measured by the PHQ-9) and/or probable PTSD (as measured by the PCL-6). The approach of creating a composite variable due to high correlation between mental health outcomes aligns with other previously published methods of analysis (e.g., in [16, 30]). A series of binary logistic regressions were then conducted which investigated univariable associations between presence of a probable common mental health disorder (AMD) and each of the predictor variables (demographics (e.g., age, ethnicity, gender), personal factors (e.g., living situation), occupational factors (e.g., length of time with organisation, whether working from home is enjoyed), resilience, productivity, wellbeing (e.g., satisfaction, happiness), and, social identity (e.g., social support, multiple identities). All data analysis was carried out using SPSS V27 [46].

### Power

An a-priori binary logistic regression power analysis was conducted on G*power 3.1 [47]. Treating AMD as the outcome, with the significance level set at 0.05, power of 0.8, H_0_ value of 0.16 (assuming a baseline prevalence of 16%, as literature reports one in six employees in the UK have a mental health condition [48]) and a H_1_ value of 0.26 (assuming a 10% increase in a COVID-19 affected sample - in line with frontline worker psychopathology prevalence derived from a COVID-19 related meta-review [49]), indicated that 523 participants were required for analysis. Multivariable binary logistic analyses were planned after univariable regressions but were not completed due to low levels of power after recruitment issues. The results below should be interpreted as preliminary pilot data which provides a snapshot of probable incidence of common mental health issues in response-focused UK civil servants during the COVID-19 pandemic, and associated factors.

## Results

In total, the survey link was clicked 246 times. 87 records were excluded due to incompletion, and a further 14 were filtered out from the survey for not meeting the eligibility criteria (i.e., due to not having experience of working from home during the COVID-19 pandemic). This resulted in an overall sample size of *n* = 145, which was below the desired power. This is discussed in more detail in the limitations.

### Sample characteristics

Table 1 displays the characteristics of the sample used within the current study. In general, the majority of respondents were female, white, between the ages of 35–44, did not live alone nor have children in the household, and were currently working on a COVID-19 focused role at the time of completing the survey.

**Table 1:** Sample characteristics and binary logistic regression results (study 1)

Characteristic	Level	Total		Employees not meeting threshold criteria for AMD		Employees meeting threshold criteria for AMD		Odds ratio (95% CI)	*P*-value
		*n*	%	Count	Percentage	Count	Percentage		
Age	18–34	35	24.1	71%	71.43%	10	29%	**3.37 (1.15 to 9.86)**	**0.026**
	35–44	44	30.3	80%	79.55%	9	20%	2.17 (0.74 to 6.34)	0.158
	45+	66	45.5	89%	89.39%	7	11%	Reference	
Ethnicity	White	134	92.4	82%	82.09%	24	18%	0.98 (0.20 to 4.84)	0.982
	Non-White	11	7.5	82%	81.82%	2	18%	Reference	
Gender	Male	41	28.5	88%	87.80%	5	12%	0.58 (0.20 to 1.66)	0.306
	Female	103	71.5	81%	80.58%	20	19%	Reference	
Lives alone	Yes	28	19.3	75%	75.00%	7	25%	1.82 (0.67 to 4.89)	0.239
	No	116	80.0	84%	84.48%	18	16%	Reference	
Lives with children	Yes	96	69.6	83%	83.33%	16	17%	1.18 (0.46 to 3.01)	0.734
	No	42	30.4	81%	80.95%	8	19%	Reference	
Currently working on COVID duties	Yes	81	55.9	84%	83.95%	13	16%	0.75 (0.32 to 1.76)	0.507
	No	64	44.1	80%	79.69%	13	20%	Reference	
Length of time with Organisation	0–2 years	59	40.7	80%	79.66%	12	20%	1.31 (0.56 to 3.10)	0.532
	2 + years	86	59.3	84%	83.72%	14	16%	Reference	
Future work arrangement preference	Home	37	25.5	89%	89.19%	4	11%	0.54 (0.17 to 1.71)	0.297
	Office	3	2.1	33%	33.33%	2	67%	8.95 (0.77 to 103.84)	0.080
	Hybrid	104	71.7	82%	81.73%	19	18%	Reference	
Resilience	Medium/High	101	69.7	93%	92.86%	1	7%	**0.24 (0.10 to 0.57)**	**0.001**
	Low	44	30.3	66%	65.91%	15	34%	Reference	
Productivity: Task performance	Low	36	24.8	67%	66.67%	12	33%	**4.19 (1.53 to 11.48)**	**0.005**
	Average	34	23.4	82%	82.35%	6	18%	1.80 (0.57 to 5.65)	0.318
	High	75	51.7	89%	89.33%	8	11%	Reference	
Productivity: Contextual Performance	Low	32	22.1	72%	71.88%	9	28%	2.35 (0.85 to 6.52)	0.101
	Average	43	29.7	84%	83.72%	7	16%	1.17 (0.41 to 3.34)	0.774
	High	70	48.3	86%	85.71%	10	14%	Reference	
Productivity: Counterproductive Work Behaviour	Low	31	21.4	94%	93.55%	2	6%	**0.17 (0.04 to 0.81)**	**0.026**
	Average	54	37.2	87%	87.04%	7	13%	**0.38 (0.14 to 1.00)**	**0.049**
	High	60	41.4	72%	71.67%	17	28%	Reference	
		**Mean**	**SD**	**n**,** mean**,** SD**		**n**,** mean**,** SD**			
Satisfied	11-point Likert-scale (0 = not at all, 10 = completely)	7.43	1.50	*n* = 115 m = 7.74 SD = 1.10		*n* = 21 m = 5.57 SD = 2.21		**0.43 (0.29 to 0.64)**	**< 0.001**
Worthwhile	11-point Likert-scale (0 = not at all, 10 = completely)	7.66	1.74	*n* = 116 m = 8.08 SD = 1.28		*n* = 23 m = 5.57 SD = 2.21		**0.40 (0.27 to 0.58)**	**< 0.001**
Happy	11-point Likert-scale (0 = not at all, 10 = completely)	7.23	1.80	*n* = 113 m = 7.55 SD = 1.48		*n* = 23 m = 5.65 SD = 2.39		**0.59 (0.46 to 0.76)**	**< 0.001**
Job satisfied	11-point Likert-scale (0 = not at all, 10 = completely)	6.45	2.29	*n* = 111 m = 6.81 SD = 2.08		*n* = 24 m = 4.79 SD = 2.50		**0.70 (0.48 to 0.85)**	**< 0.001**
Enjoy working from home	10-point Likert-scale (1 = not at all, 10 = completely)	8.12	1.89	*n* = 118 m = 8.35 SD = 1.70		*n* = 23 m = 6.96 SD = 2.40		**0.71 (0.57 to 0.89)**	**0.003**
Identification	7-point Likert-scale (1 = not at all, 7 = definitely)	5.21	1.19	*n* = 114 m = 5.23 SD = 1.49		*n* = 24 m = 5.25 SD = 1.20		0.99 (0.72 to 1.36)	0.943
Social support	7-point Likert-scale (1 = not at all, 7 = definitely)	5.27	1.37	*n* = 117 m = 5.21 SD = 1.26		*n* = 26 m = 4.98 SD = 1.29		0.83 (0.59 to 1.17)	0.291
Multiple identities	7-point Likert-scale (1 = not at all, 7 = definitely)	4.10	1.61	*n* = 115 m = 4.15 SD = 1.63		*n* = 26 m = 3.88 SD = 1.52		0.90 (0.69 to 1.18)	0.452
Identity continuity	7-point Likert-scale (1 = not at all, 7 = definitely)	4.55	1.72	*n* = 116 m = 4.52 SD = 1.80		*n* = 26 m = 4.68 SD = 1.31		1.06 (0.82 to 1.36)	0.666

### Mental health outcomes

The rates of common mental health disorders in the sample population were 15.2% (95% confidence interval (CI): 9.8-22.1%) probable moderate depression (*n* = 22), 9.7% (95% CI: 5.4-15.7%) moderate anxiety (*n* = 14), and 7.6% (95% CI: 3.9-13.2%) PTSD (*n* = 11). A total of 17.9% (95% CI: 12.1-25.2%; *n* = 26) of the sample met the threshold criteria for one or more of probable moderate or severe anxiety, moderate or severe depression, and/or PTSD (indicated by AMD).

### Risk and resilience factors

Table 1 displays the associations between presence of AMD and various demographic, professional, and personal categorical predictor variables. Significant associations indicated: employees aged between 18 and 34 were over three times more likely to experience AMD in comparison to those aged 45+; employees with higher resilience were less likely to experience AMD than those with low resilience; employees with low task performance were over four times more likely to experience AMD in comparison to those with high task performance; and, employees who reported low or average levels of counterproductive behaviour were less likely to experience AMD. Employees reporting to enjoy working from home were significantly less likely to experience AMD.

### Summary

In summary, Study 1 established that 17.9% of the sample of UK Government employees met the threshold criteria for probable moderate anxiety, moderate depression, or post-traumatic stress disorder. Univariable binary logistic regressions suggest that younger, less resilient, less productive individuals, with less enjoyment for working from home, were more likely to present with poorer mental health.

### Study 2

#### Method

##### Data

Cross-sectional secondary data analysis was conducted on data collected by one UK Government response-focused organisation (the same as in Study 1) across three time points (May, June, and August of 2020) using an online survey. The survey sought to monitor and support UK Government employee’s wellbeing during the COVID-19 incident and response within their organisation. The survey was designed to take between five to ten minutes to complete and included standardised questions to allow comparisons. It was confidential and anonymous, and distributed using internal newsletters and word of mouth (e.g., in team meetings, briefings).

### Study materials

The survey included a range of demographic and professional information alongside measures of wellbeing, and workplace support and environment.

### Measures

#### Demographic and professional information

Participants were firstly asked questions related to their age, gender, ethnicity and household location: Participants were asked to select their age from the following choices: ‘*16–24’*, ‘*25–34’*, ‘*35–44’*, ‘*45–54’*, ‘*55–64’*, ‘*65 plus*’, or ‘*Prefer not to say*’; their gender from the following choices: ‘*Male’*, ‘*Female’*, ‘*I identify in another way*’, ‘*Prefer not to say’*; their ethnicity from the following choices: ‘*Any White background’*, ‘*Any Asian background’*, ‘*Any Black background’*, ‘*Any Mixed background’*, ‘*Any other ethnic group*’, ‘*Prefer not to say’*; and the location they work from the following choices: ‘*East Midlands’*; ‘*East of England’*; ‘*London*’; ‘*North East’*; ‘*North West’*; ‘*South East’*; ‘*South West*’; ‘*West Midlands’*; ‘*Yorkshire & Humber’*; ‘*Scotland*’; ‘*Wales*’; ‘*Outside the UK’*; ‘*Other*’, or ‘*Prefer Not to Say*’.

Participants were also asked to report on whether they experience any long term physical or mental health conditions using ‘*Yes*’, ‘*No*’ or ‘*Prefer not to say*’, as well as if they are a carer (i.e., care for dependents or give help/support to any family members or others) using ‘*Yes*’, ‘*No*’ or ‘*Prefer not to say*’.

Participants were asked to also to report the way in which they were currently working from the following choices: ‘*Working solely on Covid-19*’; ‘*Working solely on Business as usual* [BAU]’; ‘*Working on a combination of BAU and Covid-19’*, or ‘*Prefer not to say’*, whether they had made use of workplace support during the Covid 19 pandemic and were provided multiple options to select from.

### Workplace support

Participants were asked to answer the following questions using an 11-point Likert scale ranging from 0 (not at all) to 10 (completely): (1) “*My line manager helps and supports me*”, (2) “*My colleagues help and support me*”.

### Working environment

Participants were asked to answer the following questions using an 5-point Likert scale ranging from 1 (strongly agree) to 5 (strongly disagree): (1) “*I have opportunities during the day to look after my physical and mental health*”, (2) “*I have an acceptable workload*”, (3) “*I am treated with respect by the people I work with*”, (4) “*I have the tools and equipment I need to do my job effectively*”, (5) “*I feel confident in using workplace technologies to connect and collaborate with colleagues*”.

### Wellbeing

Participants were asked to answer the following questions using an 11-point Likert scale ranging from 0 (not at all) to 10 (completely): (1) “*Overall*,* how satisfied are you with your life nowadays?”*, (2) “*Overall*,* how happy did you feel yesterday?”*, (3) “*Overall*,* how anxious did you feel yesterday?”*, (4) “*Overall*,* how satisfied are you with your current work responsibilities?”*. The first three listed questions are regularly used by the Office for National Statistics [37], the fourth was adapted by the survey creators to assess job satisfaction.

### Statistical analysis

Descriptive statistics were calculated for all variables. To identify risk factors for happiness, anxiety, work satisfaction and life satisfaction. a two-step binary logistic regression analysis was used. Before examining possible associations between wellbeing (i.e., happiness, anxiety, life, and work satisfaction) and predictors, several variables were recoded for analysis. For wellbeing measures, a score of > 6 was coded to indicate high life or work satisfaction and happiness, and a score of > 5 indicated anxiety, as recommended by ONS guidance [37]. Age was recoded into four groups (16–34; 35–55; 45–54 and 55+), ethnicity was recoded into two groups (White, all other ethnicities); location was recoded into two groups (London, all other locations); working role was recoded into two groups (those working on COVID-19 (i.e., solely COVID or joint with business as usual work), and those working on business as usual), all to allow a more comparable number of participants between groups. All ‘*Prefer not to say*’ and ‘*other*’ selections in the demographic and professional information were categorised as missing data for analysis (all percentages, across all three time points, can be found in Table 2). Lastly, in relation to Gender only males and females were included in analyses due to a small number of participants in ‘*I identify in another way*’ (consistently < 1% of the sample across all three time points; Table 2 provides more details). All participants completed all outcome measures fully. Following recoding, univariable binary regression was used to identify each variable that was associated with happiness, anxiety, work satisfaction and life satisfaction. Variables with a p-value < 0.25 were then included in a multivariable regression [50]; following the method of purposeful selection of covariates in logistic regression [50] that suggests that variables reaching significance at 0.25 indicate reasonable association with the outcome variable and should be retained for further analysis (e.g., as used in [51, 52]). Values in the multivariable regression models were deemed significant if ≤ 0.05.

**Table 2:** Demographics of sample (study 2)

	May		June		August	
Variable	Count	%	Count	%	Count	%
**Age**						
16–34	303	17.7	303	17.7	270	19.0
35–44	356	20.8	356	20.8	295	20.7
45–54	417	24.3	417	24.3	352	24.8
55+	321	18.7	321	18.7	267	18.8
Missing	316	18.4	316	18.4	238	16.7
***Gender***						
Female	915	64.35	1083	63.22	713	59.72
Male	272	19.13	357	20.84	257	21.52
I identify in another way	6	0.42	7	0.41	7	0.59
Prefer not to say	128	9	159	9.28	126	10.55
Missing	101	7.1	107	6.25	91	7.62
***Ethnicity***						
White	1211	70.7	1211	70.7	1047	73.6
All other ethnicities	207	12.1	207	12.1	145	10.2
Missing	295	17.2	295	17.2	230	16.2
***Civil Service grade***						
Executive office and below	303	17.7	303	17.7	265	18.6
Higher executive officer	238	13.9	238	13.9	186	13.1
Senior executive officer	346	20.2	346	20.2	285	20.0
Grade 6 and above	485	28.3	485	28.3	426	30.0
Missing	341	19.9	341	19.9	260	18.3
***Long standing health condition***						
Yes	273	19.2	291	16.99	197	16.5
No	997	70.11	1238	72.27	834	69.85
Prefer not to say	102	7.17	117	6.83	102	8.54
Missing	50	3.52	67	3.91	61	5.11
***Caring responsibilities***						
Yes	596	41.91	711	41.5	499	41.79
No	743	52.25	876	51.1	596	49.92
Prefer not to say	45	3.16	73	4.3	59	4.94
Missing	38	2.67	53	3.1	40	3.35
***Working location***						
London	544	38.3	638	37.2	544	38.3
Outside of London	843	59.3	1022	59.7	843	59.3
Missing	35	2.5	53	3.1	35	2.5
***Work type***						
COVID-19	832	58.5	1010	59.0	832	58.5
Business as usual	554	39.0	635	37.1	554	39.0
Missing	36	2.5	68	4.0	36	2.5
***Scale measures***	**M**	**SD**	**M**	**SD**	**M**	**SD**
I have opportunities during the day to look after my physical and mental health	2.43	1.17	2.49	1.14	2.65	1.1
I have an acceptable workload	2.54	1.11	2.56	1.1	2.7	1.11
I am treated with respect by the people I work with	1.9	0.98	1.87	0.89	2	0.92
I have the tools and equipment I need to do my job effectively	2.35	1.07	2.25	1	2.28	0.96
I feel confident in using workplace technologies to connect and collaborate with colleagues	2.02	0.98	1.96	0.89	2.03	0.88
My line manager helps and supports me	7.42	2.61	7.36	2.57	7.16	2.65
My colleagues help and support me	7.79	1.99	7.73	2.05	7.56	2.17

## Results

In total, 1422 participants data was analysed from the May survey, *n* = 1194 for August, *n* = 1713 for June. Demographics of the sample can be found in Table 2. Table 3 presents counts and percentages of outcomes measures in May, June, and August of 2020. In summary, life satisfaction ranged from 42.6 to 51.9% across the three time points, job satisfaction ranged from 32.7 to 51.4%, happiness from 48.1 to 52.8%, and anxiety from 35.3 to 44.9%.

**Table 3:** Counts and percentages of outcome measures in May, June and August of 2020

	May		June		August	
	Count	%	Count	%	Count	%
**Satisfied (life)**	707	49.7	889	51.9	509	42.6
**Satisfied (work)**	729	51.3	880	51.4	391	32.7
**Happy**	684	48.1	842	49.2	631	52.8
**Anxious**	502	35.3	639	37.3	536	44.9

### Univariate analyses outcomes

All univariable logistic regression outcomes for each wellbeing measure (i.e., happiness, anxiety, life satisfaction and work satisfaction) at each time point can be found in Supplementary information (Tables S1-3). All univariable associations significant at the < 0.25 level were entered into the subsequent multivariable logistic regressions.

### Multivariable analyses outcomes

Results of multivariable binary logistic regression analysis for happiness is presented in Table 4. Consistently across the three time points, employees that reported using workplace wellbeing support and those who reported less opportunities to look after their mental and physical health were more likely to be unhappy. Other variables significant at one or two of the timepoints were: work type (COVID-19 vs. business as usual), having a long standing physical or mental health condition illness or disability, having line manager help and support, and having colleague help and support.

**Table 4:** Multivariable binary logistic regression outcomes for happiness across three time points (May, June, and August of 2020)

		May						June						August					
		Happy		Unhappy		Adjusted Odds ratio	*p*	Happy		Unhappy		Adjusted Odds ratio	*p*	Happy		Unhappy		Adjusted Odds ratio	*p*
Variable	Level	*n*	%	*n*	%	(95% CI)		*n*	%	*n*	%	(95% CI)		*n*	%	*n*	%	(95% CI)	
Age	16–34	127	47	143	53	Not entered	N/A	149	49	154	51	1.10 (0.77–1.58)	0.607	79	37	133	63	1.01 (0.66–1.76)	0.756
	35–44	147	50	148	50	Not entered	N/A	180	51	176	49	0.98 (0.69–1.39)	0.911	78	33	159	67	1.19 (0.74–1.89)	0.471
	45–54	173	49	179	51	Not entered	N/A	213	51	204	49	0.95 (0.68–1.32)	0.743	87	33	179	67	1.22 (0.78–1.91)	0.394
	55+	138	52	129	48	Not entered	N/A	175	55	146	45	Reference		94	41	137	59	Reference	
Ethnicity	White	511	49	536	51	Not entered	N/A	617	51	594	49	Not entered	N/A	288	34	553	66	1.35 (0.84–2.15)	0.213
	Other	78	54	67	46	Not entered	N/A	109	53	98	47	Not entered	N/A	55	42	77	58	Reference	
Location	London	239	44	305	56	1.21 (0.91–1.58)	0.153	317	50	321	50	Not entered	N/A	143	34	284	67	Not entered	N/A
	All other	434	51	409	49	Reference		498	49	524	51	Not entered	N/A	181	32	377	68	Not entered	N/A
Work type	COVID	374	45	458	55	**1.38 (1.05–1.81)**	**0.019**	475	47	535	53	0.90 (0.70–1.15)	0.393	217	34	428	66	Not entered	N/A
	BAU	296	53	258	47	Reference		335	53	300	47	Reference		164	32	344	68	Not entered	N/A
Pay Grade	AA, EA, EO	141	53	124	47	1.20 (0.84–1.71)	0.329	156	51	147	49	Not entered	N/A	82	41	119	59	0.83 (0.52–1.31)	0.417
	HEO	94	51	92	49	0.91 (0.61–1.34)	0.627	123	52	115	48	Not entered	N/A	65	37	110	63	0.88 (0.54–1.44)	0.599
	SEO	128	45	157	55	1.21 (0.87–1.70)	0.263	172	50	174	50	Not entered	N/A	83	32	175	68	0.90 (0.58–1.40)	0.644
	G7+	207	49	219	51	Reference		242	50	243	50	Not entered	N/A	83	29	202	71	Reference	
Health condition	Yes	108	40	165	60	**1.74 (1.27–2.38)**	**0.001**	127	44	164	56	1.32 (0.97–1.79)	0.082	52	26	145	74	1.26 (0.82–1.93)	0.299
	No	512	51	485	49	Reference		648	52	590	48	Reference		308	37	526	63	Reference	
Carer	Yes	286	48	310	52	Not entered	N/A	361	51	350	49	Not entered	N/A	157	32	342	69	0.87 (0.61–1.24)	0.43
	No	359	48	384	52	Not entered	N/A	438	50	438	50	Not entered	N/A	211	35	385	65	Reference	
Used wellbeing support	None	431	51	418	49	**0.75 (0.58–0.98)**	**0.035**	582	54	488	46	**0.58 (0.45–0.74)**	**< 0.001**	294	36	515	64	**0.53 (0.372–0.77)**	**< 0.001**
	Yes	253	44	320	56	Reference		260	40	383	60	Reference		97	25	288	75	Reference	
		**M**	**SD**	**M**	**SD**			**M**	**SD**	**M**	**SD**			**M**	**SD**	**M**	**SD**		
My line manager helps and supports me *A*		7.92	2.4	6.96	2.75	**0.94 (0.88–1.01)**	**0.069**	8.01	2.18	6.73	2.76	**0.92 (0.87–0.98)**	**0.012**	7.86	2.37	6.81	2.72	0.96 (0.88–1.04)	0.327
My colleagues help and support me *A*		8.22	1.7	7.39	2.13	**0.86 (0.80–0.94)**	**0.001**	8.32	1.68	7.15	2.2	**0.77 (0.71–0.84)**	**< 0.001**	8.14	1.86	7.28	2.25	0.94 (0.85–1.05)	0.286
Opportunities to look after mental/physical health *B*		2.18	1.1	2.65	1.15	**1.24 (1.08–1.43)**	**0.003**	2.17	1.04	2.8	1.15	**1.47 (1.28–1.68)**	**< 0.001**	2.26	1	2.84	1.1	**1.48 (1.20–1.82)**	**< 0.001**
I have an acceptable workload *B*		2.31	1.0	2.75	1.14	1.01 (0.87–1.18)	0.861	2.28	0.98	2.84	1.13	1.14 (0.99–1.32)	0.075	2.39	1	2.84	1.13	1.03 (0.84–1.28)	0.765
I am treated with respect by the people I work with *B*		1.76	0.9	2.04	1	1.08 (0.86–1.18)	0.926	1.72	0.81	2.01	0.94	0.89 (0.75–1.05)	0.173	1.81	0.86	2.1	0.93	1.07 (0.84–1.37)	0.598
Tools and equipment to do my job effectively *B*		2.19	1.0	2.49	1.07	1.08 (0.92–1.26)	0.36	2.05	0.89	2.44	1.05	1.11 (0.96–1.28)	0.179	2.05	0.9	2.39	0.97	1.17 (0.91–1.49)	0.221
Confident using workplace technology to connect/collaborate *B*		1.89	1.0	2.14	0.99	1.13 (0.96–1.33)	0.151	1.82	0.82	2.09	0.93	1.06 (0.90–1.24)	0.473	1.91	0.83	2.08	0.9	1.08 (0.85–1.37)	0.516

*Please note*: All significant values are bolded. A: 10-point Likert scale (0 = not at all, 10 = completely), B: 5-point Likert scale (1 = strongly agree − 5 strongly disagree). BAU = Business as usual, AA, EA and EO = Administrative Assistants, Administrative Officers, Executive Officer. HEO = higher executive officer. SEO = senior executive officer. G7 + = Grade 7, Grade 6

Results of multivariable binary logistic regression analysis for anxiety is presented in Table 5. Consistently across the three time points, those who reported less opportunities to look after their mental and physical health were more likely to be anxious. Other variables significant at one or two of the timepoints were: ethnicity, civil service grade, having a long standing physical or mental health condition illness or disability, being a career, using workplace wellbeing support, having line manager help and support, and having colleague help and support.

**Table 5:** Multivariable binary logistic regression outcomes for anxiety across three time points (May, June, and August of 2020)

		May						June						August					
		Anxious		Not Anxious		Adjusted Odds ratio	*p*	Anxious		Not Anxious		Adjusted Odds ratio	*p*	Anxious		Not Anxious		Adjusted Odds ratio	*p*
Variable	Level	*n*	%	*n*	%	(95% CI)		*n*	%	*n*	%	(95% CI)		*n*	%	*n*	%	(95% CI)	
Age	16–34	109	40	161	60	0.69 (0.43–1.11)	0.128	123	41	180	59	0.79 (0.52–1.19)	0.262	121	57	91	43	**0.48 (0.30–0.76)**	**0.002**
	35–44	109	37	186	63	0.96 (0.61–1.50)	0.851	137	38	219	62	0.96 (0.66–1.42)	0.85	114	48	123	52	0.89 (0.57–1.37)	0.583
	45–54	122	35	230	65	0.85 (0.56–1.31)	0.467	147	35	270	65	1.10 (0.76–1.59)	0.624	147	55	119	45	0.64 (0.42–0.98)	0.039
	55+	71	27	196	73	Reference		99	31	222	69	Reference		95	41	136	59	Reference	
Ethnicity	White	374	36	673	64	**0.47 (0.29–0.78)**	**0.003**	433	36	778	64	Not entered	N/A	442	53	399	47	1.11 (0.71–1.73)	0.645
	Other	32	22	113	78	Reference		70	34	137	66	Not entered	N/A	62	47	70	53	Reference	
Gender	Male	97	36	175	64	Not entered	N/A	122	34	235	66	1.13 (0.83–1.54)	0.436	129	50	128	50	Not entered	N/A
	Female	321	35	594	65	Not entered	N/A	408	38	675	62	Reference		368	52	345	48	Not entered	N/A
Location	London	211	39	333	61	0.73 (0.533–1.00)	0.051	227	36	411	64	Not entered	N/A	233	55	194	45	Not entered	N/A
	All other	276	33	567	67	Reference		391	38	631	62	Not entered	N/A	286	51	272	49	Not entered	N/A
Work type	COVID	305	37	527	63	0.81 (0.60–1.10)	0.182	389	39	621	61	0.85 (0.64–1.11)	0.233	354	55	291	45	1.19 (0.87–1.62)	0.275
	BAU	175	32	379	68	Reference		220	35	415	65	Reference		257	51	251	49	Reference	
	AA, EA, EO	85	32	180	68	**0.62 (0.94–0.97)**	**0.038**	97	32	206	68	0.95 (0.63–1.43)	0.798	88	44	113	56	1.30 (0.83–2.01)	0.25
	HEO	65	35	121	65	0.84 (0.52–1.34)	0.46	97	41	141	59	**0.67 (0.45–1.00)**	**0.049**	91	52	84	48	1.24 (0.77–1.98)	0.377
Pay grade	SEO	110	39	175	61	0.70 (0.47–1.04)	0.077	149	43	197	57	0.74 (0.53–1.05)	0.092	137	53	121	47	1.12 (0.75–1.69)	0.577
	G7+	139	33	287	67	Reference		162	33	323	67	Reference		161	57	124	44	Reference	
Health condition	Yes	119	44	154	56	**0.60 (0.43–0.84)**	**0.003**	128	44	163	56	**0.68 (0.49–0.94)**	**0.021**	113	57	84	43	0.83 (0.56–1.22)	0.344
	No	318	32	679	68	Reference		427	34	811	66	Reference		409	49	425	51	Reference	
Carer	Yes	227	38	369	62	**0.73 (0.53–1.00)**	**0.003**	280	39	431	61	0.80 (0.60–1.05)	0.111	261	52	238	48	Not entered	N/A
	No	241	32	502	68	Reference		309	35	567	65	Reference		307	52	289	49	Not entered	N/A
Used wellbeing support	None	266	31	583	69	**1.65 (1.22–2.22)**	**0.001**	347	32	723	68	**1.45 (1.11–1.89)**	**0.006**	397	49	412	51	1.38 (1.00–1.91)	0.053
	Yes	236	41	337	59	Reference		292	45	351	55	Reference		234	61	151	39	Reference	
		**M**	**SD**	**M**	**SD**			**M**	**SD**	**M**	**SD**			**M**	**SD**	**M**	**SD**		
My line manager helps and supports me *A*		7.25	2.68	7.52	2.56	**0.92 (0.85–0.99)**	**0.032**	7.05	2.65	7.54	2.51	1.00 (0.94–1.06)	0.928	6.9	2.71	7.44	2.57	1.04 (0.96–1.13)	0.298
My colleagues help and support me *A*		7.45	2.13	7.97	1.89	**1.16 (1.05–1.27)**	**0.002**	7.45	2.16	7.89	1.96	1.08 (0.99–1.17)	0.079	7.45	2.17	7.69	2.16	0.98 (0.89–1.07)	0.606
Opportunities to look after mental/physical health *B*		2.77	1.19	2.24	1.12	**0.75 (0.64–0.88)**	**< 0.001**	2.77	1.2	2.33	1.07	**0.73 (0.63–0.85)**	**< 0.001**	2.79	1.12	2.48	1.05	**0.80 (0.67–0.96)**	**0.016**
I have an acceptable workload *B*		2.83	1.16	2.38	1.05	0.90 (0.76–1.06)	0.21	2.81	1.17	2.42	1.02	0.87 (0.74–1.02)	0.081	2.86	1.14	2.51	1.05	0.84 (0.71–1.05)	0.134
I am treated with respect by the people I work with *B*		2.07	1.05	1.81	0.93	0.94 (0.78–1.12)	0.484	1.98	0.93	1.8	0.86	0.91 (0.76–1.09)	0.299	2.07	0.93	1.93	0.89	0.94 (0.75–1.17)	0.56
Tools and equipment to do my job effectively *B*		2.52	1.11	2.25	1.03	0.91 (0.76–1.09)	0.316	2.38	1.05	2.17	0.95	1.09 (0.93–1.29)	0.273	2.38	1.01	2.16	0.88	0.97 (0.78–1.21)	0.788
Confident using workplace technology to connect/collaborate *B*		2.1	1.01	1.98	0.96	1.13 (0.94–1.37)	0.193	2.03	0.93	1.92	0.86	1.04 (0.88–1.24)	0.651	2.09	0.97	1.95	0.77	1.01 (0.81–1.25)	0.95

*Please note*: All significant values are bolded. A: 10-point Likert scale (0 = not at all, 10 = completely), B: 5-point Likert scale (1 = strongly agree − 5 strongly disagree). BAU = Business as usual, AA, EA and EO = Administrative Assistants, Administrative Officers, Executive Officer. HEO = higher executive officer. SEO = senior executive officer. G7 + = Grade 7, Grade 6

Results of multivariable binary logistic regression analysis for work satisfaction is presented in Table 6. Consistently across the three time points, those who reported to have a less acceptable workload, had less supportive line manager and colleagues, and were younger in age were more likely to be unsatisfied with work. Other variables significant at one or two of the timepoints were: ethnicity, using workplace wellbeing support, having the tolls and equipment to work effectively, and being confident in using workplace technology to connect/collaborate.

**Table 6:** Multivariable binary logistic regression outcomes for work satisfaction across three time points (May, June, and August of 2020)

		May						June						August					
		Satisfied with work		Unsatisfied		Adjusted Odds ratio	*p*	Satisfied with work		Unsatisfied		Adjusted Odds ratio	*p*	Satisfied with work		Unsatisfied		Adjusted Odds ratio	*p*
Variable	Level	*n*	%	*n*	%	(95% CI)		*n*	%	*n*	%	(95% CI)		*n*	%	*n*	%	(95% CI)	
Age	16–34	122	45	148	55	**1.84 (1.19–2.87)**	**0.007**	156	51	147	49	**1.76 (1.15–2.70)**	**0.01**	91	43	121	57	**2.52 (1.47–4.30)**	**< 0.001**
	35–44	163	55	132	45	1.18 (0.78–1.78)	0.434	200	56	156	44	1.18 (0.79–1.76)	0.412	118	50	119	50	1.10 (0.67–1.82)	0.703
	45–54	190	54	162	46	1.16 (0.78–1.73)	0.468	224	54	193	46	1.15 (0.78–1.69)	0.487	131	49	135	51	0.98 (0.61–1.58)	0.928
	55+	155	58	112	42	Reference		191	60	130	41	Reference		124	54	107	46	Reference	
Ethnicity	White	560	53	487	47	1.21 (0.80–1.85)	0.365	656	54	555	46	Not entered	N/A	383	46	458	55	**2.44 (1.42–4.19)**	**0.001**
	Other	86	59	59	41	Reference		117	57	90	43	Not entered	N/A	80	61	52	39	Reference	
Gender	Male	135	50	137	50	1.22 (0.78–1.69)	0.247	186	52	171	48	Not entered	N/A	125	49	132	51	Not entered	N/A
	Female	498	54	417	46	Reference		597	55	486	45	Not entered	N/A	347	49	366	51	Not entered	N/A
Location	London	272	50	272	50	Not entered	N/A	333	52	305	48	Not entered	N/A	185	43	242	57	1.10 (0.76–1.60)	0.616
	All other	444	53	399	47	Not entered	N/A	526	51	496	49	Not entered	N/A	264	47	294	53	Reference	
Work type	COVID	412	50	420	50	1.16 (0.86–1.57)	0.321	482	48	528	52	1.15 (0.87–1.54)	0.328	256	40	389	60	1.38 (0.97–1.96)	0.077
	BAU	303	55	251	45	Reference		367	58	268	42	Reference		267	53	241	47	Reference	
	AA, EA, EO	154	58	111	42	1.10 (0.71–1.70)	0.674	183	60	120	40	1.11 (0.73–1.68)	0.625	130	65	71	35	0.64 (0.39–1.07)	0.086
	HEO	101	54	85	46	1.36 (0.87–2.11)	0.18	124	52	114	48	1.37 (0.89–2.10)	0.152	83	47	92	53	0.95 (0.56–1.60)	0.84
Pay grade	SEO	140	49	145	51	1.06 (0.73–1.54)	0.776	182	53	164	47	0.99 (0.69–1.42)	0.961	110	43	148	57	1.04 (0.65–1.66)	0.864
	G7+	223	52	203	48	Reference		253	52	232	48	Reference		122	43	163	57	Reference	
Used wellbeing support	None	438	52	411	48	Not entered	N/A	592	55	478	45	**0.66 (0.50–0.87)**	**0.003**	374	46	435	54	1.02 (0.71–1.48)	0.907
	Yes	291	51	282	49	Not entered	N/A	288	45	355	55	Reference		162	42	223	58	Reference	
		**M**	**SD**	**M**	**SD**			**M**	**SD**	**M**	**SD**			**M**	**SD**	**M**	**SD**		
My line manager helps and supports me *A*		8.37	2.08	6.43	2.73	**0.86 (0.80–0.92)**	**< 0.001**	8.38	1.91	6.29	2.74	**0.84 (0.78–0.90)**	**< 0.001**	8.3	1.98	6.23	2.77	**0.82 (0.75–0.90)**	**< 0.001**
My colleagues help and support me *A*		8.54	1.53	7	2.12	**0.73 (0.66–0.80)**	**< 0.001**	8.52	1.5	6.9	2.21	**0.75 (0.68–0.82)**	**< 0.001**	8.46	1.52	6.83	2.33	**0.72 (0.72–0.92)**	**< 0.001**
Opportunities to look after mental/physical health *B*		2.18	1.1	2.69	1.19	0.97 (0.83–1.14)	0.728	2.17	1.01	2.83	1.18	0.99 (0.85–1.16)	0.927	2.28	0.98	2.95	1.1	1.03 (0.83–1.26)	0.818
I have an acceptable workload *B*		2.21	0.99	2.88	1.13	**2.10 (1.76–2.51)**	**< 0.001**	2.1	0.86	3.05	1.11	**2.05 (1.73–2.44)**	**< 0.001**	2.19	0.87	3.11	1.12	**1.85 (1.47–2.32)**	**< 0.001**
I am treated with respect by the people I work with *B*		1.69	0.93	2.12	0.98	1.17 (0.95–1.44)	0.14	1.62	0.75	2.13	0.95	1.15 (0.95–1.41)	0.163	1.7	0.76	2.25	0.96	1.04 (0.80–1.37)	0.758
Tools and equipment to do my job effectively *B*		2.14	1.03	2.56	1.06	1.15 (0.96–1.38)	0.124	2	0.88	2.52	1.04	1.12 (0.94–1.33)	0.197	1.9	0.71	2.58	1.02	**1.75 (1.34–2.30)**	**< 0.001**
Confident using workplace technology to connect/collaborate *B*		1.92	0.97	2.13	0.98	1.20 (0.99–1.46)	0.061	1.78	0.79	2.15	0.95	**1.25 (1.04–1.51)**	**0.018**	1.82	0.73	2.19	0.96	1.11 (0.86–1.42)	0.419

*Please note*: All significant values are bolded. A: 10-point Likert scale (0 = not at all, 10 = completely), B: 5-point Likert scale (1 = strongly agree − 5 strongly disagree). BAU = Business as usual, AA, EA and EO = Administrative Assistants, Administrative Officers, Executive Officer. HEO = higher executive officer. SEO = senior executive officer. G7 + = Grade 7, Grade 6

Results of multivariable binary logistic regression analysis for life satisfaction is presented in Table 7. Consistently across the three time points, those with less opportunities to look after their mental and physical health and those with less supportive colleagues were more likely to be unsatisfied with their life. Other variables significant at one or two of the timepoints were: age, having a having a long standing physical or mental health condition illness or disability, using workplace wellbeing support, having a support line manager and being confident in using workplace technology to connect/collaborate.

**Table 7:** Multivariable binary logistic regression outcomes for life satisfaction across three time points (May, June, and August of 2020)

		May						June						August					
		Satisfied with life		Unsatisfied		Adjusted Odds ratio	*p*	Satisfied with life		Unsatisfied		Adjusted Odds ratio	*p*	Satisfied with life		Unsatisfied		Adjusted Odds ratio	*p*
Variable	Level	*n*	%	*n*	%	(95% CI)		*n*	%	*n*	%	(95% CI)		*n*	%	*n*	%	(95% CI)	
Age	16–34	130	48	140	52	1.04 (0.674 − 1.61)	0.851	145	48	158	52	**1.51 (1.04–2.20)**	**0.031**	110	52	102	48	**0.57 (0.35–0.94)**	**0.027**
	35–44	147	50	148	50	0.88 (0.58–1.32)	0.53	184	52	172	48	1.28 (0.89–1.84)	0.186	107	45	130	55	0.79 (0.50–1.25)	0.313
	45–54	177	50	175	50	1.01 (0.69–1.49)	0.954	228	55	189	45	1.02 (0.72–1.44)	0.935	112	42	154	58	1.02 (0.66–1.59)	0.928
	55+	149	56	118	44	Reference		188	59	133	41	Reference		105	46	126	55	Reference	
Ethnicity	White	530	51	517	49	Not entered	N/A	634	52	577	48	1.34 (0.92–1.94)	0.122	378	45	463	55	1.27 (0.79–2.05)	0.326
	Other	79	54	66	46	Not entered	N/A	124	60	83	40	Reference		67	51	65	49	Reference	
Gender	Male	155	57	117	43	0.79 (0.57–1.11)	1.68	195	55	162	45	Not entered	N/A	119	46	138	54	Not entered	N/A
	Female	449	49	466	51	Reference		580	54	503	46	Not entered	N/A	332	47	381	53	Not entered	N/A
Location	London	256	47	288	53	1.06 (0.80–1.42)	0.674	338	53	300	47	Not entered	N/A	174	41	253	59	1.38 (0.98–1.95)	0.068
	All other	437	52	406	48	Reference		524	51	498	49	Not entered	N/A	240	43	318	57	Reference	
Work type	COVID	402	48	430	52	1.11 (0.84–1.49)	0.463	513	51	497	49	0.82 (0.63–1.07)	0.146	278	43	367	57	Not entered	N/A
	BAU	295	53	259	47	Reference		342	54	293	46	Reference		220	43	288	57	Not entered	N/A
Pay Grade	AA, EA, EO	139	52	126	48	1.11 (0.74–1.66)	0.625	169	56	134	44	Not entered	N/A	100	50	101	50	1.44 (0.91–2.30)	0.122
	HEO	99	53	87	47	0.84 (0.57–1.35)	0.545	131	55	107	45	Not entered	N/A	80	46	95	54	1.37 (0.84–2.23)	0.208
	SEO	131	46	154	54	1.32 (0.91–1.92)	0.147	174	50	172	50	Not entered	N/A	112	43	146	57	1.22 (0.79–1.87)	0.367
	G7+	221	52	205	48	Reference		257	53	228	47	Not entered	N/A	117	41	168	59	Reference	
Health condition	Yes	116	42	157	58	**1.58 (1.13–2.21)**	**0.007**	121	42	170	58	1.44 (1.04–1.98)	0.26	79	40	118	60	0.78 (0.52–1.18)	0.243
	No	525	53	472	47	Reference		690	56	548	44	Reference		383	46	451	54	Reference	
Carer	Yes	289	48	307	52	Not entered	N/A	383	54	328	46	Not entered	N/A	207	42	292	59	0.81 (0.57–1.14)	0.806
	No	377	51	366	49	Not entered	N/A	455	52	421	48	Not entered	N/A	271	46	325	55	Reference	
Used wellbeing support	None	438	52	411	48	0.80 (0.60–1.06)	0.118	617	58	453	42	**0.56 (0.43–0.72)**	**< 0.001**	363	45	446	55	**0.61 (0.43–0.86)**	**0.005**
	Yes	269	47	304	53	Reference		272	42	371	58	Reference		146	38	239	62	Reference	
		**M**	**SD**	**M**	**SD**			**M**	**SD**	**M**	**SD**			**M**	**SD**	**M**	**SD**		
My line manager helps and supports me *A*		7.91	2.36	6.94	2.75	**0.93 (0.86–1.00)**	**0.038**	8.04	2.12	6.62	2.8	**0.89 (0.84–0.95)**	**< 0.001**	7.92	2.25	6.59	2.79	0.94 (0.86–1.02)	0.115
My colleagues help and support me *A*		8.23	1.75	7.35	2.12	**0.88 (0.80–0.96)**	**0.004**	8.36	1.66	7.05	2.2	**0.76 (0.70–0.83)**	**< 0.001**	8.21	1.75	7.08	2.32	**0.85 (0.76–0.94)**	**0.002**
Opportunities to look after mental/physical health *B*		2.14	1.13	2.71	1.14	**1.53 (1.31–1.79)**	**< 0.001**	2.21	1.06	2.79	1.15	**1.35 (1.17–1.55)**	**< 0.001**	2.32	1.03	2.89	1.09	**1.51 (1.23–1.84)**	**< 0.001**
I have an acceptable workload *B*		2.33	1.06	2.74	1.13	0.89 (0.76–1.05)	0.175	2.31	0.99	2.84	1.14	1.11 (0.96–1.29)	0.174	2.4	1.01	2.91	1.13	1.06 (0.87–1.30)	0.56
I am treated with respect by the people I work with *B*		1.75	0.93	2.06	1.00	1.12 (0.94–1.33)	0.203	1.72	0.82	2.03	0.94	0.82 (0.69–0.98)	0.029	1.78	0.82	2.17	0.95	0.98 (0.78–1.24)	0.88
Tools and equipment to do my job effectively *B*		2.21	1.03	2.48	1.08	0.95 (0.80–1.13)	0.552	2.04	0.9	2.47	1.04	1.15 (0.99–1.35)	0.073	2.06	0.88	2.44	0.98	0.98 (0.78–1.24)	0.887
Confident using workplace technology to connect/collaborate *B*		1.89	0.97	2.15	0.97	1.10 (0.92–1.31)	0.287	1.79	0.79	2.14	0.96	**1.24 (1.05–1.47))**	**0.013**	1.88	0.82	2.13	0.91	1.25 (0.99–1.57)	0.06

*Please note*: All significant values are bolded. A: 10-point Likert scale (0 = not at all, 10 = completely), B: 5-point Likert scale (1 = strongly agree − 5 strongly disagree). BAU = Business as usual, AA, EA and EO = Administrative Assistants, Administrative Officers, Executive Officer. HEO = higher executive officer. SEO = senior executive officer. G7 + = Grade 7, Grade 6

### Summary

In summary, Study 2 found between 42.6% and 51.9% of the sample to be satisfied with their life, 32.7–51.4% to be satisfied with their work, 48.1–52.8% to be happy, and 35.3–44.9% to be anxious across all three surveyed timepoints. The most consistent factor associated with better mental health across all three time points in three of the outcome measures, was those who reported more opportunities to look after their mental and physical health.

## Discussion

This study sought to estimate the rate of mental health disorders in a novel population of UK emergency response civil servants who had experience of working from home during COVID-19, as well as to provide information on related risk and resilience factors. Study 1 established a total of 17.9% of the sample met the threshold criteria for probable moderate anxiety, moderate depression, or post-traumatic stress disorder (data collected May – August of 2022). Younger, less resilient, less productive individuals, with lower personal wellbeing and less enjoyment for working from home, were more likely to present with poorer mental health. Study 2 found between 42.6% and 51.9% of the sample to be satisfied with their life, 32.7–51.4% to be satisfied with their work, 48.1–52.8% to be happy, and 35.3–44.9% to be anxious across all three surveyed timepoints (May, June, and August of 2020). The most consistent factor associated with better wellbeing across all three time points in three of the outcome measures, was those who reported more opportunities to look after their mental and physical health.

The authors believe this paper to be the first to examine the rate of UK emergency response civil servants during the COVID-19 pandemic. In the current study, a total of 17.9% of the sample met the threshold criteria for probable moderate anxiety, moderate depression, or PTSD collectively. At a more granular level, 15.2% met the threshold for probable depression, 9.7% anxiety, and 7.6% PTSD, suggesting this study found enhanced rates in comparison to standard pre-COVID UK estimates [53]. However, more recent reports published by Public Health England [48] (now known as the UK Health Security Agency) suggest that one in six employees (~ 16%) in the workplace suffer with common mental health disorders, which is in line with findings from the current research.

The impact of the COVID-19 pandemic on mental health is extremely topical. For example, a recent systematic review and meta-analysis sought to report prevalence of depression, anxiety, insomnia, posttraumatic stress disorder, and psychological distress among COVID-19 affected populations. A total of 55 studies were included and a prevalence rate of 16.0% was reported for depression, 15.2% for anxiety, and 21.9% for PTSD [53], similar to the rates found in the current study.

In terms of specific frontline occupations, greater prevalence of mental health disorders whilst working through the COVID-19 pandemic has been shown in: UK frontline health and social care workers (e.g., 58% met the threshold for probable clinical significance for anxiety, depression or PTSD) [18]; intensive care unit staff (e.g., 45% met the threshold for probable clinical significance for severe depression, PTSD, severe anxiety, or problem drinking) [30]; and teachers (e.g., anxiety (17%), depression (19%), and stress (30%) [17]. In summary, the current findings report lower rates in comparison to other well documented frontline occupations during COVID-19, but are marginally higher in comparison to the prevalence of common mental disorders in the workplace [48]. This slight elevation could reflect that working from home on the frontline raises new challenges that may be associated with increased mental health concern (e.g., lack of social connection or blurred boundaries [7]), but not to the same level as challenges within face-to-face frontline occupations during the pandemic due to the nature of the work and responsibilities. For example, witnessing suffering, or death of, patients within frontline hospital or care settings has been linked to negative impacts on mental wellbeing both pre [54] and during the pandemic [55], and is a challenge those working from home were unlikely to face.

In relation to risk and resilience factors, we found that younger employees were more likely to experience a mental disorder. The significant association could be explained by that working during the pandemic, and contributing to the COVID-19 response, may have been the first time working on emergency response-based work for many younger staff. A recent paper documented mental health outcomes among civil servants aiding in COVID-19 control in China. Using the PHQ-9 and GAD-7, akin to the current study, in a total of 867 participants, 37% and 38% met the threshold criteria for depression and anxiety, respectively [56]. This research found being younger, and having fewer years of work experience, were associated with poorer mental health outcomes [56], which supports the findings of the current research.

Additionally, we found that UK Civil Servants staff who reported lower resilience, personal wellbeing, productivity, or job satisfaction were more likely to report poorer mental health. In the wider literature, there are well documented relationships between resilience [57], productivity [58], job satisfaction [59] and mental health which support the findings of the current study. As a result, in this occupational context, it is recommended that workplaces should be seeking to continually build and improve employee resilience, essentially ensuring employees have necessary resources and skills to support themselves and others. For example, employees could seek to bolster resilience using social activities to increase social ties and support networks [13]. Furthermore, staff could be monitored and checked in on in terms of job satisfaction and productivity to ensure they are performing for the organisation, and this translates to good wellbeing.

Study 2 highlights the importance of this, as having supportive line managers and colleagues were associated with higher levels of wellbeing, across multiple time points and for multiple outcome measures. That is to say that improving social bonds between team members, ensuring that supervisors feel confident to identify potential mental health difficulties, and communicate comfortably with staff about them, whilst fostering a culture of mutual respect could be a key focus of organisational resilience enhancement [60, 61]. Our results also suggest that employers and staff should be proactive in supporting those who are younger and those who are seemingly less productive (e.g., not meeting performance goals or are displaying counterproductive work behaviour). Those who enjoy working from home were also less likely to have poor mental health whilst working from home, employers and organisations should seek to break common barriers to working from home and ensure the positive aspects are maximised to increase staff experience [62].

The current paper also found non-significant associations for whether participants were actively working on the COVID-19 response, which suggests that actively responding to COVID-19 was not, in itself, a specific factor influencing staff mental health. This finding is also supported by recent research [56] which also found no difference between frontline and non-frontline workers both in depression and anxiety severity among civil servants. It is suggested that due to secondments and staff movement to aid the pandemic response, the BAU roles became busier due to teams operating with reduced numbers of staff but still needed to meet the same targets, in essence non-responding civil servants also experienced a rise in workload and demands [56].

### Limitations

Despite being the first paper (to the authors’ knowledge) to establish rates of mental health issues using standardised and validated measures in a sample of UK Government response employees, the research is not without limitation. For Study 1 specifically, despite exhausting possible survey distribution routes, gaining engagement with a busy taskforce was difficult, resulting in a small, underpowered sample. Secondly, the data was collected between May and August 2022, when the pandemic response was beginning to ‘wind down’ (i.e., less COVID-19 cases, lower work demands), meaning that some individuals who had been working on the COVID-19 response may have left the organisation (either due to contracts ending, or potentially if they had negative experiences, akin to the healthy worker effect [63]) which also suggests a potential bias in the sample; Thirdly, this data is cross-sectional; measuring and tracking mental health incidence longitudinally would provide more robust findings, as well as aid with inferring causation. Fourthly, it is important to consider that the survey did not collected data on when exactly employees were working during the pandemic (e.g., during lockdowns, virus surges). We suggest that future research examining wellbeing during public health emergencies should be longitudinal in method as this would allow for examinations over time where additional factors (such as external factors like virus prevalence, and restrictions) could be included in analyses. The authors believe that many limitations associated with Study 1 are addressed by Study 2; as the data used in the secondary data analysis consisted of a large sample of participants, collected during the height of the COVID-19 pandemic across multiple time points. Unfortunately, Study 2 did not use standardised mental health measures (as used in the first study) and instead used wellbeing measures; however, wellbeing is reported in the literature as being closely linked with, and a key feature of mental health [64]. Additionally, Study 2 data did not provide the opportunity to restrict to different occupations within the one select government organisation participants were from. However, the organisation is response-focused, and as noted in the discussion (in relation to Study 1 findings), it is suggested that even business as usual roles became busier due to teams operating with reduced numbers of staff (e.g., due to secondments, staff movement), suggesting that the impact of the COVID-19 pandemic could be felt by all staff.

In summary, Study 2 sought to overcome the difficulties and limitations of Study 1. Study 1 provided a cross-sectional insight into response-focused civil servants mental health and wellbeing experiences as they were exiting the COVID-19 period. The authors acknowledge the caveats apparent with Study 1. Study 2 instead provided cross-sectional snapshots of wellbeing in civil servants collected across three time periods (during the heightened pandemic), allowing for concurrent evaluation of employee wellbeing alongside understanding change over time – and identification of consistent influential factors over time. Combined, this research provides the first clear estimates of common mental health disorders in the UK Government frontline employees, using standardised and validated measures, as well as associated risk and resilience factors.

## Conclusion

The rates of common mental health disorders in home working frontline UK civil servants during the COVID-19 pandemic were lower in comparison to other well documented frontline occupations during the pandemic [17, 18, 30], but remain slightly higher in comparison to the rates of common mental disorders in the workplace [48]. Younger, less resilient, less productive individuals, with lower personal wellbeing and less enjoyment for working from home, were more likely to present with poorer mental health outcomes. As were those without opportunities to look after their physical and mental health, or those without supportive line managers and colleagues. As a result, it is important to ensuring civil servants psychological needs are met whilst responding to enhanced incidents, such as the COVID-19 pandemic.

### Electronic supplementary material

Below is the link to the electronic supplementary material.

Supplementary Material 1

## Data Availability

Participants and data owners did not consent to the full dataset being made available to the public; as a result the datasets used and/or analysed during the current study will not be publicly available. Data output files are available from the corresponding author upon reasonable request.
